# The Convenience of Single Homology Arm Donor DNA and CRISPR/Cas9-Nickase for Targeted Insertion of Long DNA Fragment

**DOI:** 10.22074/cellj.2016.4719

**Published:** 2016-09-26

**Authors:** Mohsen Basiri, Mehrdad Behmanesh, Yaser Tahamtani, Keynoosh Khalooghi, Azadeh Moradmand, Hossein Baharvand

**Affiliations:** 1 Department of Genetics, Faculty of Biological Sciences, Tarbiat Modares University, Tehran, Iran; 2 Department of Stem Cells and Developmental Biology, Cell Science Research Center, Royan Institute for Stem Cell Biology and Technology, ACECR, Tehran, Iran; 3 Department of Developmental Biology, University of Science and Culture, Tehran, Iran

**Keywords:** CRISPR-Cas Systems, Gene Targeting, Embryonic Stem Cells, Pdx1

## Abstract

**Objective:**

CRISPR/Cas9 technology provides a powerful tool for targeted modification of genomes. In this system, a donor DNA harboring two flanking homology arms is mostly used for targeted insertion of long exogenous DNA. Here, we introduced an alternative design for the donor DNA by incorporation of a single short homology arm into a circular plasmid.

**Materials and Methods:**

In this experimental study, single homology arm donor was applied along with a single guide RNA (sgRNA) specific to the homology region, and either Cas9 or its mutant nickase variant (Cas9n). Using *Pdx1* gene as the target locus the functionality of this system was evaluated in MIN6 cell line and murine embryonic stem cells (ESCs).

**Results:**

Both wild type Cas9 and Cas9n could conduct the knock-in process with this system. We successfully applied this strategy with Cas9n for generation of *Pdx1*^*GFP*^ knock-in mouse ESC lines. Altogether, our results demonstrated that a combination of a single homology arm donor, a single guide RNA and Cas9n is capable of precisely incorporating DNA fragments of multiple kilo base pairs into the targeted genomic locus.

**Conclusion:**

While taking advantage of low off-target mutagenesis of the Cas9n, our new design strategy may facilitate the targeting process. Consequently, this strategy can be applied in knock-in or insertional inactivation studies.

## Introduction

Harnessing the clustered regularly interspaced short palindromic repeats (CRISPR) and the CRISPR-associated protein (Cas) system has provided a new means, CRISPR/Cas9 technology, for introduction of targeted changes into a genome sequence (1,2). In this technology a nucleoprotein complex that consists of the Cas9 protein and a single guide RNA (sgRNA) are used to generate a double-strand break (DSB) in a specific genomic target site, determined by the sgRNA sequence (1,3). Predominantly, DSBs are repaired through the error-prone non-homologous end joining (NHEJ) mechanism which usually results in indel mutations (4). However, in the presence of a customdesigned homologous donor DNA, homology directed repair (HDR) can introduce customized changes into the DSB site (5,6). Concerns about the CRISPR/Cas9-induced off-target mutations have led to the development of a mutant Cas9 variant, Cas9 nickase (Cas9n), which makes a singlestrand break or nick in target DNA (2). Despite its lower off-target mutation rate, Cas9n was shown to be less efficient than the wild type variant (3,7). This issue has been addressed through a double nicking strategy which entails using a pair of sgRNAs along with Cas9n. This strategy was applied successfully for both gene targeting in cultured cells (7,8) and generation of mutant organisms (9). However, designing two sgRNAs with the required criteria for double nicking strategy and their co-expression in target cells might reduce the simplicity and versatility of this method. 

Using appropriate donor DNA constructs along with the CRISPR/Cas9 system has led to the efficient introduction of a variety of subtle to multiple kilobase-pair (Kbp) modifications into eukaryotic genomes (1,2,10,11). This design strategy for targeted integration of long DNA fragments into a genome is based on the application of two flanking homology arms. It has been demonstrated that 200-400 base pair (bp) homology arms can effectively be used with the CRISPR/Cas9 system (12,13) which are far shorter than multiple Kbp arms that are suggested for conventional gene targeting vectors (14). 

Here we described an alternative design for CRISPR/Cas9 mediated insertion of a long DNA fragment into the mammalian genome. In this design, we used a circular donor DNA that contained a single 318 bp homology arm in combination with Cas9n and a single sgRNA. This approach was applied to insert a green fluorescent protein (GFP) coding sequence (CDS) into the genomic locus of the pancreatic and duodenal homeobox 1 (*Pdx1*) gene in the mouse insulinoma cell line (MIN6) and mouse embryonic stem cells (ESCs). 

## Materials and Methods

### Cell culture

This experimental study was conducted on MIN6 insulinoma cell line and mouse ESCs. We cultured MIN6 cells in Dulbecco’s modified Eeagle’s medium (DMEM) that contained 5 mM glucose (Life Technologies, Germany) with 2 mM Glutamax (Life Technologies) and 10% (v/v) fetal bovine serum (Life Technologies). An undifferentiated Royan-B20 mouse ESC line (15) was maintained on gelatin-coated dishes in knock-out DMEM with 15% (v/v) knock-out serum replacement, 2 mM Glutamax, and 1x nonessential amino acids (all from Life Technologies), supplemented with 100 µM beta mercaptoethanol, 1000 U/mL recombinant leukemia inhibitory factor (16), 1 µM PD0325901 (Stemgent, USA), and 10 µM SB431542 (Sigma-Aldrich, USA) in R2i medium (15,17). 

### Genetic constructs

In order to construct the donor plasmid, a previously constructed plasmid (pEGFPN1-Pdx1, GenBank accession number: KU341334) was used, which had been resulted from insertion of an 8 Kbp mouse *Pdx1* upstream genomic sequence into pEGFPN1 vector. pEGFPN1-Pdx1 was digested with AseI and SacI (Thermo Scientific, Germany) to remove the *Pdx1* upstream fraction. The remaining fragment was blunted and recirculated with self-ligation. The resultant plasmid, pKI-Pdx1 (GenBank accession number: KU341331), harbored a 318 bp fragment from the *Pdx1 5´* untranslated region and upstream sequences were used as the single homology arm donor. A *Pdx1*-specific sgRNA (sgPdx1) which targets 157 bp upstream of *Pdx1* CDS was designed using the online CRISPR Design tool (http://crisp.mit.edu) (3). In order to construct Cas9/sgPdx1 (pCas9-sgPdx1, GenBank accession number: KU341332) and Cas9n/sgPdx1 (pCas9n-sgPdx1, GenBank accession number: KU341333) expressing plasmids, we synthesized, annealed and cloned two oligonucleotides (Table 1) into the BpiI sites of pX330 and pX335 (gifts from Feng Zhang, Addgene plasmids # 42335 and # 42335) (2) respectively, and confirmed them with sequencing (Pishgam, Iran). 

### Transfection of MIN6 cells and flow cytometry

MIN6 cells was seeded at a density of 10^4^ cells per cm^2^ in 6-well cell culture plates, 24 hours before transfection. Transfection was performed using Lipofectamin 3000 (Life Technologies, Germany) according to the manufacturer’s instructions. Briefly, 1.5 µg of each plasmid DNA (donor plasmid and Cas9/Cas9n expressing construct) and 6 µL of Lipofectamin 3000 were used per each well. The transfection medium was replaced with fresh medium after 12 hours. After 48 hours of transfection, transfected MIN6 cells were dissociated with trypsin and washed with phosphate-buffered saline. An untransfected sample was included as the negative control. Single cell suspensions of live cells were transferred into flow cytometry tubes where approximately 20000 cells per sample were acquired by a Partec PAS flow cytometer (Partec, Germany) and analyzed using FlowJo 7.6.1 software (Tree Star Inc., USA). Transfection experiments was performed in three separated biological replicates. 

### Transgenesis and genotyping of mouse embryonic stem cells

To target *Pdx1* gene, we used Royan B20 ESC line, previously evaluated in terms of pluripotency and germ line transmission (15). Approximately 10^7^ ESCs were co-transfected with 20 µg of pCas9n-sgPdx1 and 40 µg of pKI-Pdx1 by electroporation. Transfected cells were spread into two, 10 cm cell culture plates and treated with 500 µg/ mL of G418 (Sigma-Aldrich, USA) for two weeks. Antibiotic resistant colonies were picked up and cultured in multi-well plates. Genomic DNAs were extracted with a Genomic DNA extraction kit (Bioneer, Daejeon, Korea); genotyping polymerase chain reactions (PCRs) were performed with two sets of genotyping primer pairs (Table 1) and a Taq DNA Polymerase Master Mix (Ampliqon, Denmark). Each set of primers amplified the flanking genomic regions of the knock-in allele. PCR condition was as follow: 95˚C for 10 minutes, 30 cycles of 95˚C for 30 seconds, 62˚C for 30 sec5 onds, and 72˚C for 1 minute. Positive clones for both genotyping PCRs were considered as targeted clones and their PCR products were purified with a PCR product purification kit (Roche, Germany) and sequenced (Pishgam, Iran) using the same primers. Electrophoresis of the PCR products were performed in a AgaroPower electrophoresis instrument (Bioneer, Korea) on 1 % agarose gel under a 7 V per cm electric field. 

**Table 1:** Oligonucleotides and primers


Description of application	Name	Sequence	Length (nucleotides)

Construction of sgPdx1 expressing plasmid	sgPdx1 sense	CACCGGAGAACTGTCAAAGCGATC	24
	sgPdx1 antisense	AAACGATCGCTTTGACAGTTCTCC	24
Genotyping and sequencing of knock-in and wild type alleles	F1	TTGCAGGCCAGCCAGGCTAC	20
	R1	TCAGGGTGGTCACGAGGGTG	20
	F2	GTCCTGTCGGGTTTCGCCAC	20
	R2	TCCCTGCTCCAGTGATCCCA	20
qPCR quantification of GFP copy number	GFP-F	ACGACGGCAACTACAAGAC	19
	GFP-R	TTGATGCCGTTCTTCTGCTT	20
qPCR quantification of *Fgf10* copy number	*Fgf10*-F	TTTGGTGTCTTCGTTCCCTGT	21
	*Fgf10*-R	TAGCTCCGCACATGCCTTC	19
qPCR quantification of *Sry* copy number	*Sry*-F	CATTTATGGTGTGGTCCCGT	20
	*Sry*-R	ATCTTCAATCTCTGTGCCTCCTG	23


### Quantification of transgene copy number

Real-time quantitative PCR (qPCR) was applied to quantify transgene copy numbers in ESC lines. For this purpose, we extracted genomic DNAs from each cell line as described above. Tenfold serial dilutions of each genomic DNA sample were prepared in nuclease-free water and applied as the template in qPCRs. Three sets of qPCR reactions were performed using primer pairs (Table 1) specific for GFP (representing the transgene), *Sry* (single copy endogenous target) and *Fgf10* (double copy endogenous target). qPCR was conducted with 2 μL of the diluted DNA in duplicate on a Rotor-Gene 6000 Real-time Thermal Cycler (Corbett Research Pty. Ltd., Australia). Acquired quantification cycles (Cqs) were applied for calculation of efficiency and copy numbers as described previously (18,20). Briefly, we calculated amplification efficiencies to ensure that the method’s requirements (amplification efficiency >90%) were met. Acquired Cqs in each dilution were normalized by the respective Cqs of *Sry*. GFP copy numbers were determined relative to respective internal controls, *Sry* and *Fgf10*, using the comparative Cq method (∆∆Cq). Transgene copy number were estimated with seven qPCR replicates for each transgenic cell line. 

### Statistical analysis

Statistical analysis was performed with GraphPad Prism 6 (GraphPad Software, Inc., San Diego, CA, USA) through the use of two-way analysis of variances (ANOVA) and Tukey’s multiple comparison test at 5% level of significance. Data were presented as mean ± SD. 

## Results

### *Pdx1* gene targeting in MIN6 cells

**Fig.1:**
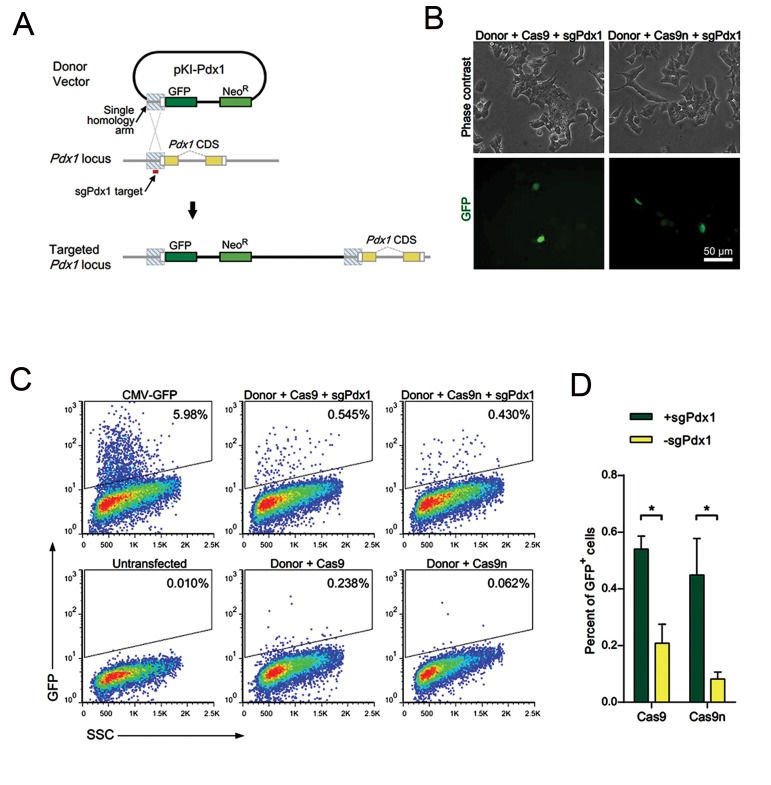
RNA guided gene targeting with a single homology arm donor plasmid in the MIN6 cell line. A. Schematic illustration of the vector design and targeted locus. Proper targeting is expected to place the green fluorescent protein (GFP) coding sequence (CDS) under the control of the endogenous *Pdx1* promoter, B. Fluorescent microscope images of MIN6 cells co-transfected with the donor (pKI-Pdx1) in combination with Cas9/sgPdx1 (pCas9-sgPdx1) or Cas9n/sgPdx1 (pCas9n-sgPdx1) expressing plasmids, C. Flow cytometry analysis of MIN6 cells transfected with different combinations of donor, Cas9/sgPdx1 and Cas9n/sgPdx1 expressing plasmids. Control samples were transfected with Cas9 or Cas9n expressing vectors which contained a BpiI site instead of sgPdx1. A control experiment with a CMV-GFP expressing plasmid showed total transfection efficiency. The untransfected control was used as a blank, and D. The percentage of GFP-expressing MIN6 cells transfected with donor and Cas9 or Cas9n expressing plasmids in the presence or absence of sgPdx1. *; Determines significant differences (P<0.05). Error bars represent SD (n=3).

In order to evaluate the feasibility of single homology arm for gene knock-in, we used a donor plasmid (pKI-Pdx1) that harbored a single homology region specific to the mouse *Pdx1* locus and a GFP CDS. Introduction of a sgRNA guided singleor double-strand break upstream of the *Pdx1* CDS and subsequent HDR within the homology region were intended to result in the insertion of whole donor vector into the *Pdx1* locus, flanked with two identical copies of the homology arm sequence (Fig.1A). 

We have used insulinoma MIN6 cells which constantly express *Pdx1*, for convenient detection of knock-in events. In these cells, targeted insertion of GFP sequence into the *Pdx1* locus is expected to result in GFP expression. After co-transfection of MIN6 cells with combinations of donor plasmid DNA and either sgPdx1-Cas9 or sgPdx1Cas9n expressing plasmids, we have observed GFP+ cells (Fig.1B). A control experiment with a constitutive GFP expressing plasmid showed the relatively low efficiency (approximately 6 %) of the transfection procedure in MIN6 cells (Fig.1C). However, both Cas9 and Cas9n mediated targeting led to significantly (P<0.05) higher frequencies of GFP+ cells when compared with corresponding control groups that lacked sgPdx1 (Fig.1C,D). Under these settings we did not observe a significant difference (P=0.53) between efficiencies of Cas9 and Cas9n (Fig.1D). Of note, the difference between Cas9 and Cas9n in the absence of sgPdx1 was not significant (P=0.21). These results suggest that both Cas9 and Cas9n could mediate the knock-in process with the single homology arm donor plasmid at comparable efficiencies. 

### Generation of *Pdx1^GFP^* knock-in embryonic stem cell lines

**Fig.2:**
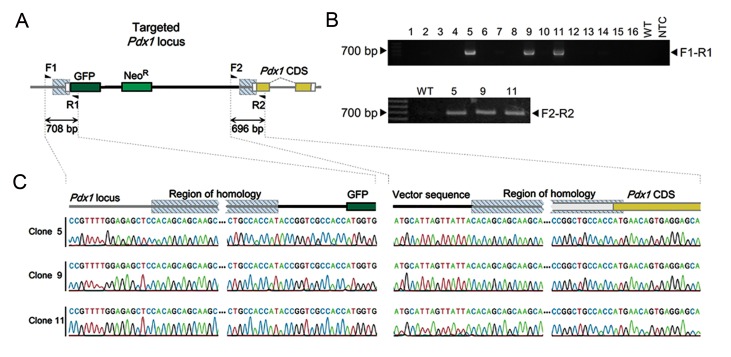
Generation of knock-in mouse ESCs by a single homology arm donor plasmid. A. Schematic presentation of targeted locus and genotyping primers, B. PCR genotyping of 16 ESC colonies transfected with the donor and Cas9n/sgPdx1 expressing plasmids, selected by G418 treatment. Three positive colonies for F1-R1 genotyping were tested by F2-R2 primers and C. Sequencing results for F1-R1 (left) and F2-R2 (right) PCR products from all three positive clones. ESCs; Embryonic stem cells and PCR; Polymerase chain reaction.

**Fig.3:**
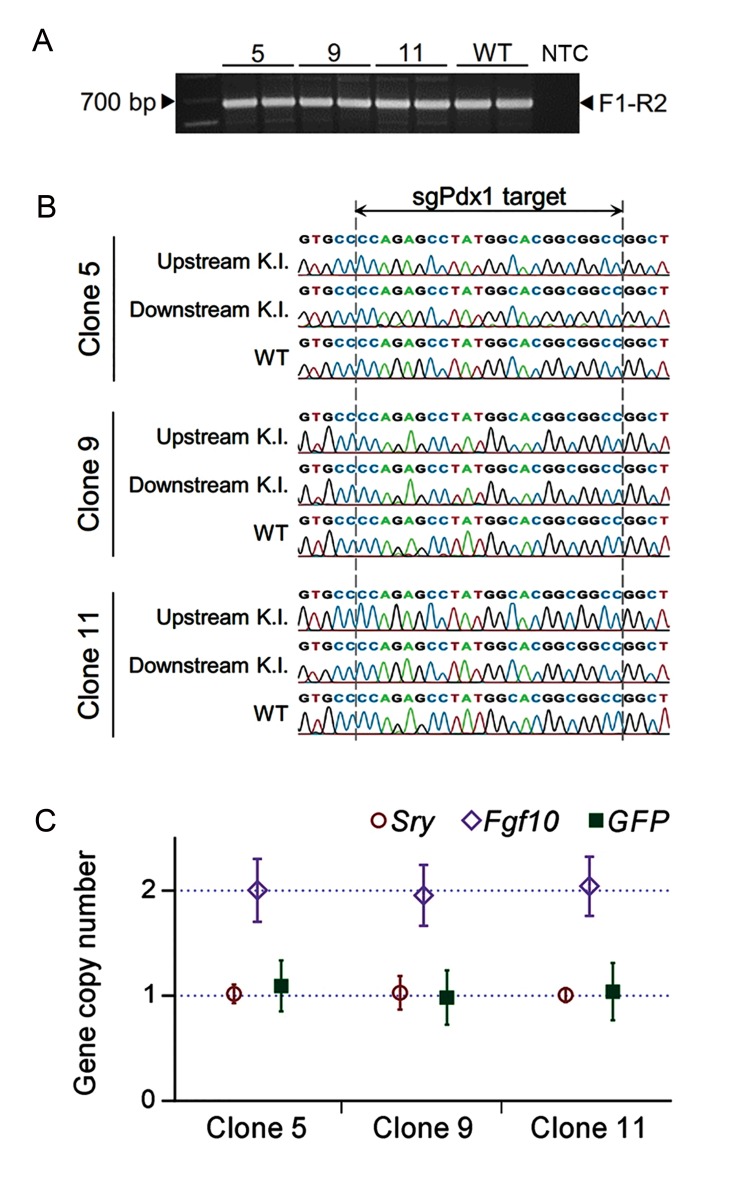
Examining the knock-in ESC clones in terms of zygosity of the *Pdx1* locus, subtle mutations in the sgPdx1 target, and random integration of the transgene. A. PCR genotyping of three targeted loci showed that all contained a wild type allele, B. Sequences of sgPdx1 targets in both sides of the targeted allele and the wild type allele contained no mutations and C. Copy number quantification with qPCR demonstrated that all three clones contained only one copy of the GFP sequence when compared with the single copy (hemizygous) *Sry* locus and the double copy *Fgf10* locus. Copy numbers were calculated relative to the mean of *Sry*. Data are presented as mean ± SD with n=7. K.I.; Targeted knock-in allele, WT; Wild type allele, ESCs; Embryonic stem cells, and qPCR; Quantitative polymerase chain reaction.

Generation of *Pdx1^GFP^* knock-in embryonic stem cell lines Given the utility of knock-in ESCs for generation of transgenic animals and differentiation studies, we aimed to evaluate the combination of single homology arm donor and Cas9n for generation of *Pdx1^GFP^* knock-in mESC lines. Resultant antibiotic resistant ESC colonies were screened by two PCR genotyping primer pairs specific to the desired targeted allele (Fig.2A). Among 16 colonies subjected to PCR genotyping, 3 were positive for both PCR reactions (Fig.2B). Further investigation of the knock-in alleles with DNA sequencing revealed that the flanking sequences of homology arm regions were identical to predicted targeted allele in all three knock-in clones (Fig.2C). PCR genotyping for wild type allele showed that all three knock-in cell lines were heterozygous in *Pdx1* locus (*Pdx1^GFP/+^*),harboring a wild type *Pdx1* allele (Fig.3A). Sequences of wild type and targeted alleles revealed no mutation at the nicking site (Fig.3B). A qPCR-based assay results indicated that only one copy of the GFP gene existed in each knock-in cell line (Fig.3C) which ensured the absence of randomly integrated copies of the donor vector. 

## Discussion

Here we demonstrated the feasibility of a single homology arm design for CRISPR/Cas9-targeted insertion of a long DNA fragment into a mammalian genome. This strategy simplified the design and cloning procedure of the donor construct by using only a single, short homology arm. Our results showed that a single homology arm donor along with a single sgRNA and Cas9n could be applied for targeted insertion of long DNA fragments into the mammalian genome. We successfully applied this strategy to generate knock-in Pdx1^GFP^ ESC lines, the genomic sequences of which revealed the precise integration of the donor vector into the genomic target. 

Although the CRISPR/Cas9 system provides an efficient way for gene targeting, the off-target activity of the system remains an issue of concern. Nicking on a single genomic target can improve safety at the expense of efficiency (3,7). Interestingly, we did not observe any significant difference between the efficiencies of Cas9 and Cas9n when used along with the single homology arm donor in MIN6 cells. However, these results might be affected by low transfection efficiency in our experimental setting. Further investigations with different sgRNAs and cell types would be required to validate these findings. Nevertheless, we have successfully applied Cas9n with our single homology arm donor for generation of knock-in ESCs with a frequency of 18.75% (3 targeted out of 16 antibiotic resistant colonies). According to the performance of Cas9n in MIN6 cells, we used this nickase variant in ESCs which was expected to decrease the chance of off-target mutations. Consistent with this expectation, we observed indel mutation neither in the sgPdx1 target sites, nor in the targeted locus or wild type allele. Although extensive off-target analyses were not conducted in this study, Cas9n has repeatedly been shown to be less mutagenic than wild type Cas9 in previous studies (2,3,7,8,12). 

Introduction of two nearby nicks with a pair of sgRNAs may increase both efficiency and specificity (7,8), but this process requires the design, construction and co-transfection of two sgRNAs which should meet a number of criteria for optimal activity (7,21). Our strategy, in contrast, minimizes the design complexity by using a single sgRNA and a short, single homology arm. Hypothetically, using a single homology arm not only simplifies the design, but also may increase efficiency by reducing the number of homologous recombination events required for vector integration. A similar principle has been previously applied to design conventional long homology arm insertional targeting vectors (14). However, more investigations are required to compare the single versus double homology arm designs in terms of efficiency. A possible drawback of using single homology arm is the insertion of total backbone of the donor vector into the genome. This can be amended by either using minicircle vectors or post-targeting deletion of the vector sequences using site specific recombinase strategies such as Cre-lox system. 

The single homology arm used in this study contained the sgPdx1 target sequence. Therefore, the homology arm could be subjected to Cas9 or Cas9n activity which might lead to cutting or nicking the donor vector. Further investigations would be required to confirm the occurrence of the nick in the donor vector and its impact on targeting efficiency. However, previous studies on long homology arms showed that both nicking and DSB in the homology arm increase targeting efficiency (22). 

## Conclusion

The proposed design for the donor DNA has provided a convenient means for RNA guided gene targeting. Although further studies are required for comprehensive evaluation of targeting efficiency and specificity, here we have demonstrated a proof of principle for the single homology arm design strategy. The simplicity and adequate efficiency for derivation of knock-in cell lines may favor the use of this design strategy, particularly for clonal gene targeting in cells such as pluripotent stem cells. 
